# miR-223: An Immune Regulator in Infectious Disorders

**DOI:** 10.3389/fimmu.2021.781815

**Published:** 2021-12-10

**Authors:** Shun Yuan, Qi Wu, Zhiwei Wang, Yanjia Che, Sihao Zheng, Yuanyang Chen, Xiaohan Zhong, Feng Shi

**Affiliations:** Department of Cardiovascular Surgery, Renmin Hospital of Wuhan University, Wuhan, China

**Keywords:** MiR-223, hematopoietic differentiation, immunity, activation, infectious diseases, biomarker

## Abstract

MicroRNAs (miRNAs) are diminutive noncoding RNAs that can influence disease development and progression by post-transcriptionally regulating gene expression. The anti-inflammatory miRNA, miR-223, was first identified as a regulator of myelopoietic differentiation in 2003. This miR-223 exhibits multiple regulatory functions in the immune response, and abnormal expression of miR-223 is shown to be associated with multiple infectious diseases, including viral hepatitis, human immunodeficiency virus type 1 (HIV-1), and tuberculosis (TB) by influencing neutrophil infiltration, macrophage function, dendritic cell (DC) maturation and inflammasome activation. This review summarizes the current understanding of miR-223 physiopathology and highlights the molecular mechanism by which miR-223 regulates immune responses to infectious diseases and how it may be targeted for diagnosis and treatment.

## Introduction

MicroRNAs (miRNAs) are small, non-coding RNAs comprising 20–25 nucleotides that play a critical role in post-transcriptional regulation of gene expression by binding target messenger RNA (mRNA) (1–3). As a powerful regulator of gene expression, miRNAs have become a research focus in the medical domain. Since their discovery in 1993, an increasing number of miRNAs are shown to have a profound biological impact (4). According to miR-Base 16, more than 15,000 miRNA gene loci in over 140 species and more than 17,000 distinct mature miRNA sequences have been discovered to date (5). Approximately 1,881 precursors and 2,588 mature miRNA sequences have been found in humans, responsible for the expression of more than 60% of genes (6, 7). Despite the variety of miRNAs, all miRNA regulate mRNA in a similar way. Indeed, miRNAs contain a seed region at the 5’-end which specifically binds to the 3’untranslated region (3’UTR) of the mRNA (8). miRNAs are highly conserved in evolution and share a significant degree of homology between species. There is growing evidence that miRNAs are powerful regulators of normal physiology, including body metabolism and immunity, tissue growth and development, and cell multiplication and apoptosis (9). Recent studies have identified several miRNAs, called inflammation-related miRNAs, that regulate immune responses. For example, miR-21 levels increase in macrophages infected with mycobacterium by attenuating the inflammatory response by targeting toll-like receptor 4 (TLR4) (10). MiR-155 also mediates inflammatory responses by increasing in macrophages following stimulation of TLR9 and mediating activation of M1 polarization by targeting the c-Jun N-terminal kinase (JNK) pathway (11). Of the known miRNAs, miR-223 is a key factor in the evolution and homeostasis of the immune system, strongly regulating particular inflammatory responses (12). To date, dysregulation of miR-223 is associated with many inflammatory diseases, including myocarditis, type II diabetes, atherosclerosis, acute lung injury (ALI), rheumatoid arthritis, infections, and inflammatory bowel disease (IBD) (13–19). This review discusses new insights into the biogenesis and biological functions of miR-223, highlights its role in hematopoiesis and immune regulation, especially in response to infection, and explores a potential role for miR-223 in the diagnosis and treatment of infection.

## MiRNA Biosynthesis and Function

### MiRNA Biosynthesis and Mature

**Figure 1:**
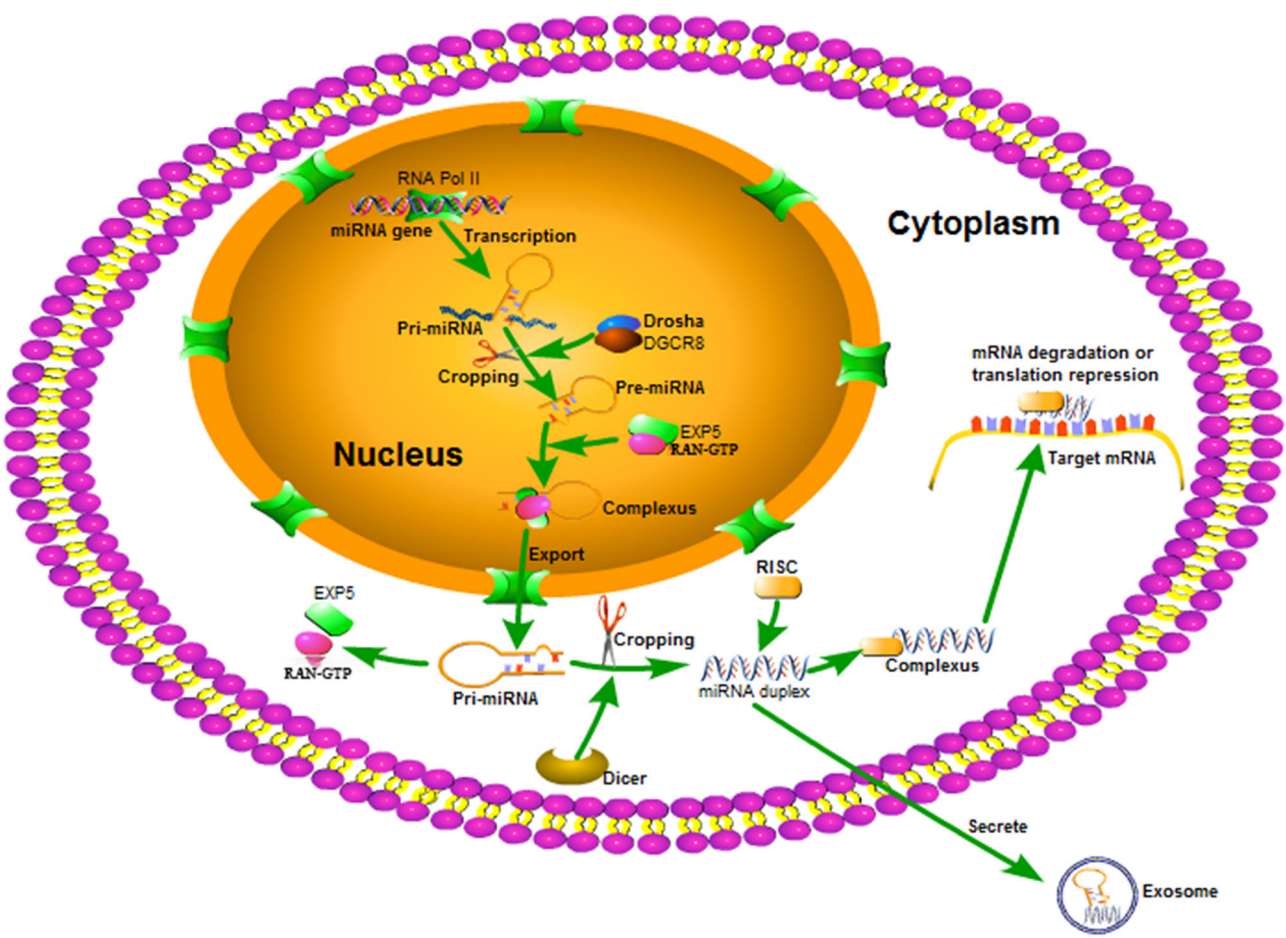
The biosynthesis and maturing process of miRNA. miRNA is transcribed as a single pri-miRNA with the help of RNA Pol II, and is then cropped into a hairpin-structured pre-miRNA through the DGCR8 complex in the nucleus. Exportin 5 and RAN-GTP aid to transferring the pre-miRNA to the cytoplasm. In the cytoplasm, pre-miRNA is processed into a miRNA duplex by the Dicer complex. One strand of the miRNA duplex bonds to RISC and guides it to various mRNA targets to regulate gene expression.

MiRNAs are encoded by miRNA genes with the help of miRNA enzymes and tightly controlled by cellular localization. While many miRNAs exist in different tissues and cells, biosynthesis and maturation follow a similar process. MiRNAs are initially transcribed in the cell nucleus as long primary miRNAs (pri-miRNAs) with the help of RNA polymerase II (20). Pri-miRNA is then cleaved by RNase II endonuclease III Drosha/DiGeorge syndrome chromosomal region 8 (DGCR8) complex to produce precursor miRNA (pre-miRNA) (21). Pre-miRNAs are transferred to the cytoplasm by Exportin-5 and Ran-GTP (22) where another specific RNase III Dicer-1 further processes pre-miRNAs into double-stranded mature miRNAs (23). After these are produced, one of the miRNA strands recognizes the RNA-induced silencing complex (RISC) and forms a complex. Mature miRNAs lead it to specific target mRNAs using base pairing to degrade mRNAs or inhibit their translation capacity (24) (**Figure 1**).

### MiRNA Function

MiRNAs are strong regulators of physiological processes such as organ development and metabolism, tissue repair and remodeling, and cell growth and differentiation by cleaving mRNAs and/or preventing gene expression (25). MiRNA can specifically recognize target mRNA using the miRNA seed, which binds specifically to the 3'-UTR of the target mRNAs (26). The seed region plays a central role in inhibiting miRNAs and is specifically recognized using effective target-forecasting tools (27). However, recent studies show that miRNA specifically recognizes the mRNA by targeting the 5’-UTR sites of mRNA to inhibit gene expression (28, 29). Research indicates that the mutual binding sequence between miRNAs and mRNAs is located in the mRNA coding sequence (30). In summary, the mRNA-miRNA interaction is a complex and multi-mechanistic process that requires further investigation.

Interestingly, miRNAs can also induce target gene expression by regulating transcription or translation. In 2008, Place et al. (31) reported that miR-373 is a transcription activator that targets the promoter region and enhances expression of cold-shock domain-containing protein C2 (CSDC2). Since this discovery, the mechanism by which miRNA induces activation (miRNAa) has elicited the attention of researchers. In 2012, studies indicated that miR-744 also regulates transcription, inducing Cyclin B1 (Ccnb1) gene transcription by binding to its promoter (32). In a follow-up study, Turner et al. showed that miRNA also regulates transcription in nematodes. The study found that miRNA lin-4 played a critical role in triggering its own transcription by binding to the lin-4-complementary element (LCE) on its promoter (33). Research has also shown that miR-558 facilitates typical miRNA gene transcription (34). In neuroblastoma cells, miR-558 linked to the promoter of heparinase and facilitated gene transcription. MiRNAs are involved in the complex processes of transcription and translation required to control target gene expression. However, there is an urgent need to elucidate whether miRNAs are involved in other steps of gene expression, such as posttranslational modification.

## Role of MiR-223 in Coding Gene Location and Regulating Transcription

**Figure 2:**
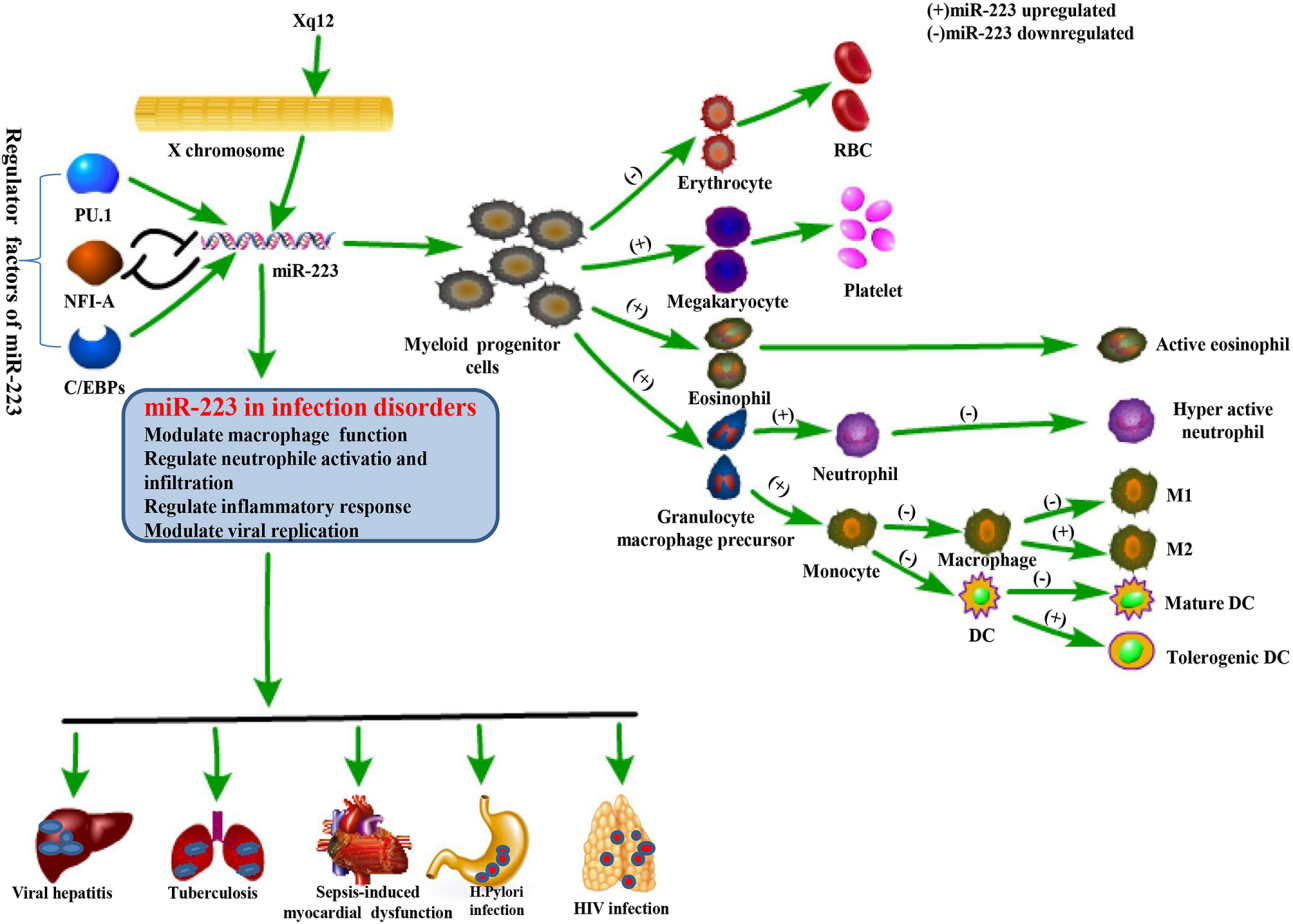
Physiological function of miR-223 and its role in regulating infectious diseases. The miR-223 gene is located on the X chromosome and modulated by several transcription factors including PU.1, C/EBPs, and NFI-A. miR-223 is a powerful regulator in hematopoietic differentiation and immune responses, and is also involved in the pathogenesis of infectious diseases by affecting macrophage function, neutrophil activation and infiltration, inflammation, and even viral replication.

MiR-233 exists on the X chromosome protein, is highly expresses in the myeloid lineage, and plays a critical role in myeloid cell differentiation (35). This gene is highly conserved in evolution and governed by an independent promoter not related to any known genes (4, 36). Myeloid transcription factors such as PU.1, CCAAT-enhancer-binding proteins (C/EBP)-α and-ß, and nuclear factor I-A (NFI-A) are shown to be powerful regulators of miR-223 expression in the hematopoietic lineage (**Figure 2**). More precisely, in osteoclast differentiation, PU.1 plays a strong role in miR-223 regulation by enhancing its promoter activities. This helps to induce osteoclastogenesis by mediating osteoclast-specific protein expression (37). C/EBP-α also facilitates miR-223 expression by binding to its promoter, thus promoting granulocyte differentiation (38). In addition, C/EBP-α strongly enhances miR-223 promoter activity with the help of PU.1 (39). In contrast, NFI-A, a negative regulator of miR-223, inhibits miR-223 expression by linking to its promoter, preventing the differentiation of granulocytes and osteoclasts (36, 37). Surprisingly, miR-223 also regulates NFI-A and C/EBP-β expression, forming negative feedback loops between the target genes and their homologous miRNAs. More precisely, miR-223, identified as an NFI-A transcription factor, binds to the NFI-A promoter and silences its transcriptional activity (38, 40, 41).

Peroxisome proliferator-activated receptor γ (PPARγ), a nuclear transcription factor, is also a key miR-223 transcription factor in macrophages. PPARγ controls miR-223 expression by directly linking to PPARγ regulatory elements (PPREs) located on the pre-miR-223 promoter and inducing M2 activation (42). In contrast, Kruppel-like factor 6 (KLF-6) is a transcription factor that prevents miR-223 expression in macrophages (43) by occupying its promoter and attenuating proinflammatory gene expression. These data indicate that miR-223 expression is controlled by different transcription factors in each tissue and cell type. Abnormal miR-223 expression may be associated with dysregulation of transcription factors that leads to tissue and cell dysfunction (44, 45).

Epigenetic mechanisms are changes in gene expression based on non-sequence alterations such as DNA methylation and chromatin conformational changes. As miRNAs are generated, epigenetic mechanisms play a critical role in miRNA expression. Common acute myeloid leukemia-associated fusion protein, AML1/ETO oncoprotein, prevents miR-223 expression by recruiting histone deacetylase (HDAC) and DNA methyltransferases to the pre-miR-223 gene and causes leukemia pathogenesis (46). Epigenetic mechanisms are also involved in miRNA regulation. Research shows that sulfatide is a powerful regulator of miR-223 in hepatocellular carcinoma cells (HCC), regulating miR-223 gene expression by preventing recruitment of acetylated histone H3 to its promoter and resulting in increased migration of HCC (47). In addition, a pivotal study found that semi-miRNAs (smiRNAs) are involved in regulating miRNA activity (48). SmiRNAs are processed from their corresponding pre-miRN by a Dicer complex. Natural miRNA anti-sense RNAs can eliminate miRNA function using base pair complementarity. The semi-miRNA derived from miR-223 (smiR-223) was found to powerfully regulate miR-223’s ability to repress target gene expression. Together, these data indicate that miR-223 transcription is a complex progress that is regulated by diverse mechanisms.

## Physiological Function of miR-223

### Role for miR-223 in Differentiation of Hematopoietic Lineages

MiR-223 is highly expressed in the hematopoietic system and is an important modulator of hematopoietic differentiation by orchestrating expression of hematopoietic stem cells, erythroid cells, and granulocyte-monocyte progenitors at different stages of development (12). Studies show that miR-223 is a powerful regulator of granulopoiesis and is expressed most highly in granulocytes. MiR-223 is upregulated during retinoic acid (RA)-induced granulocytic differentiation. Inhibiting miR-223 expression reduces the efficiency of RA-induced differentiation and induces differentiation of granulocyte-monocyte progenitors into monocytes (38, 49, 50). NFI-A, a site-specific DNA-binding protein, plays an important role in RA-induced granulopoiesis through epigenetic silencing. During RA-induced myeloid differentiation, NFI-A decreases miR-223 expression while C/EBP-α increases miR-223, preventing NFI-A expression and initiating a positive feedback loop to control miR-223 and induce granulocyte differentiation (38).

In the myeloid lineage, miR-223 is closely associated with transcription factors involved in myeloid cell differentiation, suggesting that this process would not occur without it. Surprisingly, researchers found that myeloid differentiation was not impacted in an miR-223^-/Y^ mouse model and granulocyte numbers were increased. Lack of miR-223 induced Mef2c expression, a transcription factor that promoted proliferation of granulocyte-monocyte progenitors. Indeed, in miR-223^-/Y^ animals, the specific knockout of Mef2c in myeloid cells, resulted in normal granulocyte numbers (12). Hence, miR-223 appears to be a core factor in mediating all stages of granulopoiesis.

MiR-223 is also involved in monocyte differentiation into macrophages. Studies show that miR-223 expression decreases and IκB kinase subunit-α (IKK-α) expression increases as differentiation occurs. This results in higher IKK-α protein expression, which induces p52 and impairs NF-κB signaling pathways (51). Additional studies have supported a role for the miR-223-NF-κB axis in mediating macrophage differentiation. This was shown by increasing or decreasing iR-223 expression in RAW 264.7 cells by transfecting them with pre-miR-223 or anti-miR-223, respectively. The cells were then induced to differentiate into a macrophage type1 or a macrophage type2 phenotype under different conditions. Results showed that while miR-223 upregulation was associated with a significant decrease in NF-kB expression, miR-223 downregulation led to a further decrease in NF-kB in RAW 264.7 cells and directed them towards the macrophage type1 phenotype. Conversely, when RAW 264.7 cells were directed towards the macrophage type2 phenotype, miR-223 upregulation led to a significant increase of NF-kB while miR-223 downregulation led to a further decrease in NF-kB. These data indicate that miR-223 plays a critical role in modulating the spatiotemporal activation of NF-κB and is a key regulator of macrophage plasticity (52).

Another study found that miR-223 expression was downregulated during macrophage differentiation. Lower levels of miR-223 were found to increase nucleotide-binding oligomerization domain-like receptor protein 3 (NLRP3) expression, which helps primary human monocytes (CD14^+ve^ cells) to differentiate into macrophages (53). Additional research showed that miR-223 is essential to macrophage development. Indeed, miR-223-rich microvesicles facilitated naïve THP-1 and monocyte differentiation into macrophages. Research showed that this process was impaired when miR-223 expression was prevented using an antagomiR inhibitor. These findings further demonstrate that miR-223 plays an important role in macrophage development (54). In summary, although miR-223 is downregulated during macrophage differentiation, it is essential for macrophage development, indicating that regulation of miR-223 in macrophage differentiation is a complex process.

MiR-223 is also involved to erythroid and megakaryocytic differentiation (hematopoietic differentiation). In K562 cells, an erythroid–megakaryocyte cell line, researchers found that miR-223 exhibited a powerful ability to inhibit erythroid cells and promote megakaryocyte differentiation (55, 56). A key factor in regulating erythroid and megakaryocytic differentiation, LIM domain only 2 (LIMO2), is the target for miR-223. During erythroid differentiation of K562 cells, miR-223 downregulation enhanced LMO2 expression, thus inducing erythroid development, while miR-223 upregulation impaired LMO2 expression, resulting in megakaryocytic differentiation.

MiR-223 is a critical regulatory element for eosinophil development by influencing the proliferation and differentiation of eosinophil progenitor cells. Studies show that upregulation of miR-223 induces eosinophil differentiation by promoting expression of insulin-like growth factor-1 receptor (IGF-1R). More importantly, ablation of the miR-223 gene in eosinophil progenitors resulted in an increased number of eosinophil progenitors and a delay in differentiation (57). MiR-223 expression was also upregulated in eosinophilic esophagitis and strongly correlated with esophageal eosinophil numbers, indicating that miR-223 is associated with eosinophil proliferation and differentiation (58). Thus, upregulation of miR-223 appears to play a central role in facilitating eosinophil maturation. However, a potential role for miR-223 as a therapeutic target for eosinophilic diseases requires further investigation of the mechanism by which it regulates eosinophil development.

MiR-223 also modulates DC differentiation *via* several pathways. DCs develop from hematopoietic stem cells (HSCs) and are major antigen-presenting cells that capture and present antigen to T lymphocytes. DC development is a complicated process regulated by several factors (59), including miR-223. Pioneering research discovered that miR-223 levels fluctuate when mouse HSCs differentiate into DCs *in vitro*, indicating that DC differentiation is likely impacted by miR-223 (60). A subsequent study demonstrated that miR-223 could maintain intestinal homeostasis by regulating development of intestinal DCs. In colitis models, mice with miR-223 deficiency had higher levels of monocyte-derived DCs (moDCs), which led to more severe disease. That study also found that miR-223-deficient monocytes produce more moDCs *in vitro*. Bioinformatics research has shown that miR-223 is a regulatory element of C/EBP-β, which may be how DC differentiation is regulated during colitis (61). A recent study found that miR-223 was upregulated when human embryonic stem (ES) cells were induced to differentiate into DCs *in vitro*. Overexpression of miR-223 in ES cells increased the efficiency by which this process occurred. It is shown that miR-223 directly targets TGF-β type III receptor (TGFBR3) during differentiation of ES cells into DCs (62). In summary, miR-223 is closely linked to DC differentiation by activating specific target genes which are core regulators of tissue inflammation and autoimmune disease.

MiR-223 has a powerful function in coordinating myeloid progenitor cell development and differentiation. It is upregulated by myeloid transcription factors during the development of granulocytes, megakaryocytes, and eosinophils and downregulated during differentiation of erythrocytes, macrophages, and DCs (**Table 1**). The specific mechanism by which miR-223 expression impacts myeloid differentiation requires further investigation (**Figure 2**).

**Table 1:** Targets for miR-223 in the immune system.

Target gene	Disease	Cell type	Conclusion	Reference
Mef2c	_	Granulocyte-monocyte progenitor	Promote granulocyte-monocyte progenitor proliferation	(12)
IIK-α	_	Macrophage	Promote monocyte-derived macrophage differentiation	(51)
LMO_2_	_	Erythroid	Promote erythroid differentiation	(56)
IGF-1R	_	Eosinophil progenitor	Promote eosinophil progenitor proliferation and suppress eosinophil differentiation	(57)
Pknox1	AT inflammation, CVB3-induced viral myocarditis, and cutaneous wound	Macrophage	Promote M1 polarization and inhibit M2 polarization	(63–65)
Rasa1/NFAT5	Sepsis	Macrophage	Promote M1 polarization and inhibit M2 polarization	(42, 66)
TRAF6	Viral myocarditis	Macrophage	Promote M1 polarization and inhibit M2 polarization	(67)
STAT3	_	Macrophage	Promote macrophage activation and IL-6 and IL-1β secretion	(68)
RhoB	ALI	Macrophage	Promote macrophage activation and TNF-α, IL-6 and IL-1β production	(69)
IRAK-1	Helicobacter pylori-infection	Macrophage	Promote macrophage activation and TNF-α, IL-6 and IL-1β production	(70)
NLRP3	_	Macrophage	Promote macrophage activation and IL-1β production	(71)
NLRP3	ALI	Neutrophil	Promote neutrophils activation and IL-1β production	(72)
CXCL2	TB	Neutrophil	Promote neutrophils recruitment	(73)
IKK-α	DILI	Neutrophil	Promote neutrophils activation and inflammatory responses	(75)
C/EBP-β	Colitis	DC	Promote monocyte-derived DCs differentiation and DCs towards pro-inflammatory phenotype	(61)
NLRP3	Autoimmune myocarditis	DC	Promote DCs activation and mature	(17)
RhoB	–	DC	Promote monocyte-derived DCs differentiation and DCs towards pro-inflammatory phenotype	(81)
IRAK1	Heart transplantation	DC	Promote monocyte-derived DCs differentiation and DCs towards pro-inflammatory phenotype	(82)
TGFBR3	–	DC	Increase the efficiency of ES cells to DCs differentiation	(62)

### MiR-223 Expression During the Immune Response

The activation of immune cells plays an important role in regulating the immune response. As an anti-inflammatory miRNA, miR-223 is involved in modulating immune responses by regulating the activation of immune cells such as macrophages, neutrophils, and DCs (**Table 1**).

### MiR-223 in Macrophages

MiR-223 is a powerful regulator of macrophage functions like polarization and activation. For example, miR-223 plays a critical role in modulating macrophage polarization during diet-induced adipose tissue (AT) inflammation (63). MiR-223 facilitates the differentiation of macrophages into the alternative M2 anti-inflammatory phenotype and inhibits macrophage infiltration helping to alleviate AT inflammation caused by a high-fat diet. MiR-223^-/Y^ animals have higher numbers of classically activated pro-inflammatory phenotype (M1) macrophages associated with AT inflammation, which leads to severe insulin resistance and AT disorder. MiR-223 induces macrophages towards the alternative M2 phenotype by suppressing Pknox1 expression, which improves macrophage-mediated AT inflammation and insulin resistance. A role for miR-223-induced Pknox1 expression was also shown in the coxsackievirus B3 (CVB3)-induced viral myocarditis mice model (64). In CVB3-infected mice, miR-223 decreased in heart tissue and in heart-infiltrating macrophages. Increasing miR-223 expression induced M2 macrophage development by restraining Pknox1 expression, thus mitigating the inflammatory response and reducing myocardial injury. An additional study further explored a role for the miR-223-Pknox1 axis in macrophage polarization. Mesenchymal stem cell (MSCs) transplantation results in high levels of exosome-miR-223 secretion and promotes cutaneous wound healing. Exosome-miR-223 may be ingested by macrophages, and induced to differentiate into M2 macrophages by controlling Pknox1 gene expression, thus accelerating wound healing (65).

Research has also shown that miR-223 has a powerful ability to regulate macrophage polarization. The nuclear transcription factor, PPARγ, binds directly to the pre-miR-223 promoter and induces miR-223 gene expression. Macrophages are then polarized towards an alternative M2 anti-inflammatory phenotype by targeting Rasa1 and NFAT5 (42). A separate study showed a role for miR-223-Rasal/NFAT5 axis in mediating macrophage polarization during sepsis. Plasma miR-223 levels were positively correlated with a higher proportion of M2 macrophages. MiR-223 was found to alleviate sepsis by binding to the mRNA of NFAT5 and Rasa1 and causing IL-4-meditated M2 macrophage differentiation (66). Moreover, in the viral myocarditis (VMC) mouse model, long non-coding RNA (lncRNA) maternally expressed 3 (MEG3) expression is high and miR-223 expression is low. It is shown that low lncRNA MEG3 facilitates miR-223 expression in macrophages, inhibiting M1and promoting M2 macrophage polarization. On a molecular level, upregulated miR-223 expression inhibits TNF receptor-associated factor 6 (TRAF6) and impairs NF-κB signaling, thus ameliorating VMC (67). In short, miR-223 is a powerful regulator of macrophage polarization by targeting Pknox1/Rasal/NFAT5/TRAF6 during inflammation, and up-regulating miR-223-induced polarization of macrophages towards an M2 anti-inflammatory phenotype.

MiR-223 is also involved in macrophage activation using several mechanisms. Many studies have shown that miR-223 inhibits inflammation. A low level of miR-223 is present in macrophages that are stimulated with TLR ligands, and downregulated expression of miR-223 facilitates production of proinflammatory factors such as IL-6 and IL-1β, through binding to the target gene signal transducer and activator of transcription 3 (STAT3) (68). Interestingly, IL-6 is shown to down-regulate miR-223, thus establishing a positive feedback loop for promoting IL-6 and IL-1b expression. In contrast, overexpression of miR-223 suppresses STAT3, which results in lower pro-inflammatory cytokine production. Thus, miR-223 might mediate macrophage activation by regulating the IL-6-STAT3 pathway. Consistent with previous studies, LPS stimulation reduces miR-223 production in macrophages. Further research shows that a low level of miR-223 facilitates production of the Ras homolog gene family, member B (RhoB), which enhances NF-κB and mitogen-activated protein kinases (MAPK) signaling and results in production of TNF-α, IL-6 and IL-1β (69). It is also shown that miR-223 prevents IL-1 receptor-associated kinases-1(IRAK-1) in *H. pylori*-infected macrophages. Over expression of microRNA-223 reduced NF-kB activation and TNF-α, IL-6 and IL-1β production by inhibiting IRAK-1 in *H. pylori*-infected macrophages (70). In addition, miR-223 negatively regulates inflammasome activity by preventing expression of the NLRP3, and inhibiting macrophage activation (71). In murine macrophages, a high level of miR-223 inhibits NLRP3 and IL-1b production. In summary, miR-223 inhibits macrophage function by targeting STAT3, RhoB, IRAK-1, and NLRP3, thus preventing pro-inflammatory cytokine production in different inflammatory diseases models.

### miR-223 in Neutrophils

Neutrophils are innate immune cells that play a critical role in regulating the early stages of inflammation. MiR-223 prevents neutrophil activation and chemotaxis during many inflammatory disorders. In 2008, researchers first discovered a role for miR-233 in neutrophil function using the miR-223^-/y^ mice model (12). In miR-223^-/y^ mice, an increased number of neutrophils was observed in the bone marrow, blood, and lungs, and these neutrophils exhibited hyperactive characteristics that promoted a pro-inflammatory response to disease. An additional study illustrated that miR-223 inhibits NLRP3, resulting in decreased IL-1β production and reduced inflammation (71). The miR-223-NLRP3 axis was also shown during ALI. MiR-223^−/+^ mice with ALI suffered more severe symptoms accompanied by a decreased ratio of arterial oxygen partial pressure to fractional inspired oxygen (PaO2/FiO2), an increased ratio of lung wet-to-dry weight ratio, and more cells infiltrating the airway. Overexpression of miR-223 reversed those symptoms by reducing Ly6G^+^ neutrophil numbers and inhibiting NLRP3 inflammasome activity (72). MiR-223 was also shown to modulate the immune response to tuberculosis (TB). Blood and lung tissues from humans and mice with TB contained higher levels of miR-223. Overexpression of miR-223 was found to control neutrophil recruitment to the lungs during TB infection by directly impairing expression of the chemoattractants, CXCL2 and CCL3, in myeloid cells, while knock-down of miR-223 in mice resulted in lethal neutrophil-driven inflammation during TB infection (73). In short, miR-223 controls inflammation during TB but has no impact on the bacterial killing ability of macrophages and neutrophils.

MiR-223 is also known to play a key role during the pathophysiology of hepatic injury by regulating neutrophil activation and function. In the APAP-induced hepatic injury (DILI) mice model, a high level of miR-223 was observed, helping to alleviate neutrophil activation and mitigate liver injury (74). During DILI, mitochondrial DNA (mtDNA) from damaged hepatocytes activates TLR9 on neutrophils to induce an inflammatory cascade and further injure the liver. However, TLR9 activation also induces miR-223 expression by facilitating NF-κB-induced miR-223 promoter activation. Interestingly, miR-223 can also attenuate the inflammatory response by preventing IKK-α expression and reducing NF-κB signaling, thus inducing a negative feedback loop that terminates the acute neutrophilic response (75). Similar to the APAP overdose model, a high level of miR-223 was observed in the serum and neutrophils of ethanol-fed mice. Furthermore, miR-223^-/y^ mice experienced more severe alcohol-induced liver injury and increased hepatic neutrophil infiltration and ROS and IL-6 production, suggesting that miR-223 is a key regulatory factor in mediating neutrophil activity during hepatic inflammation (76).

In summary, miR-223 appears to be abundant in neutrophils, serving to inhibit neutrophil activation and function during inflammatory diseases. Thus, miR-223- mediated neutrophil activation may be a target for treatments aimed at reducing neutrophil-driven inflammation to prevent hyperactive immune responses.

## Role of miR-223 in DC function

DCs are potent antigen-presenting cells that play a pivotal role in inducing and maintaining tolerance and immunity (77). In response to insult, DCs begin expressing high levels of MHC and costimulatory molecules and secreting pro-inflammatory cytokines that can induce excessive inflammation (78). MiR-223 negatively regulates DCs activation in response to many inflammatory diseases. In response to LPS stimulation of DCs, miR-223 expression was significantly decreased. Conversely, miR-223 expression is increased in tolerogenic DCs that are generated in response to glucocorticoid DEX and the prototypic anti-inflammatory cytokine, IL-10, during differentiation bone marrow cells into BMDCs. High levels of miR-223 help to maintain DCs in a tolerogenic state by downregulating Rasa1, Kras, and Cflar (79).

MiR-223 also plays a role in regulating Langerhans cells (LCs), skin-residential dendritic cells (80). High miR-223 expression was observed in epidermal LCs and BMDCs. Knock-down expression of miR-223 made no difference to LC maturation markers like MHC-II, CD80, and CD86, but significantly enhanced the ability of LCs to facilitate antigen-specific CD8^+^ T cell development. These data indicate that miR-223 is a negative regulator of LCs cross-presentation capacity, however the mechanism by which this occurs is not fully understood. MiR-223 was also found to regulate DC differentiation and function during colitis by directly targeting C/EBP-β (61). Indeed, in a mouse model of colitis, a low level of miR-223 existed in colonic DC subsets. MiR-223-deficient monocytes generated more moDCs and exhibited a strong pro-inflammatory phenotype that was associated with increased DC infiltration and production of proinflammatory cytokines like IL-23, IL-1b, TNF-a and IL-6 in the intestine, which led to more severe colitis.

MiR-223 plays a key role in mediating homeostasis within the intestine and may therefore be a potential target for IBD treatment. A role for miR-223 in inhibiting DC activation was also shown during autoimmune myocarditis (EAM) (17). In an EAM mouse model, overexpression of in DC impaired production of NLRP3 and promoted the polarization of DCs towards a tolerogenic DC phenotype, preventing further heart injury. During α-myosin H-chain peptide-induced EAM, the transfer of DCs that overexpressed miR-223, attenuated EAM by inducing regulatory T cell (Treg) differentiation and causing immune tolerance. Data has also shown that miR-223 controls DC activation by targeting RhoB. Overexpression of miR-223 impairs antigen uptake and presentation by BMDC and facilitates Treg cell development by inhibiting RhoB. MiR-223 also targets RhoB to induce the tolerogenic DC phenotype following OVA treatment (81). Recent studies identified a function for miR-223 in regulating DC function in a mouse heart transplant model. A high level of miR-223 was observed in the serum and in DCs from humans and mice following heart transplantation. High miR-223 levels facilitated tolerogenic DC differentiation by targeting IRAK1 *in vitro* (82). However, other studies have shown that miR-223 enhances DC maturation during experimental autoimmune encephalomyelitis (EAE) (83, 84).

Global miR-223 knockout (miR-223^-/-^) mice with EAE exhibited lower central nervous system (CNS) autoimmune inflammation and less pro-inflammatory APC infiltration than wild-type mice. Basal and LPS-induced IL-12 and IL-23 levels were also lower in DCs from miR223^-/-^ mice than wild-type mice. These results appear to contrast with studies indicating that miR-223 inhibits DC activation. It is evident that miR-223 plays a key role during cellular development, particularly in myeloid differentiation and immune cell development and function. Studies show that miR-223 levels vary between different stages of bone marrow cell development. MiR-223 deficiency impairs the development of bone marrow derived cells and inhibits DC function (85), however the exact mechanism by which this occurs requires further investigation. In summary, miR-223 is a negative regulator of DC activation, maintaining a maturation-resistant protolerogenic state during inflammation. MiR-223 may thus serve as a target for the treatment of inflammatory diseases.

### MiR-223 in the Pathophysiology of Infectious Disorders

The immune system was shown to protect its host from infectious microorganisms over a century ago. Pathogen-associated molecular patterns (PAMPs) are specifically recognized by host innate pattern recognition receptors (PRRs), including TLRs, nucleotide-binding domain leucine-rich repeat-containing proteins (NLRs), and C-type lectins (86). This process activates innate immune cells like macrophages, DCs and neutrophils, and induces them to secrete cytokines needed to prevent pathogenic microorganisms from harming the host and establish long-term protection against subsequent infection. The inflammatory response to PAMPs must be effectively controlled to eliminate pathogens while avoiding the consequences of excessive inflammation, septic shock, and cancer.

MiR-223 plays a strong role in regulating responses to infectious diseases like viral hepatitis, HIV-1, TB, *Helicobacter pylori* (*H. pylori*) infection, and sepsis, helping to maintain equilibrium between protective immune responses and host injury caused by excessive inflammation (**Table 2**). This occurs due to miR-223’s ability to mediate macrophage and neutrophil function and inflammasome activation during pathological conditions. Many inflammatory diseases are difficult to diagnose and cure due to clinical heterogeneity and a lack of specific diagnostic markers and therapeutic methods. MiRNAs are novel, sensitive, and noninvasive biomarkers and may serve as potential targets for treatment of inflammatory disorders due to their central role in regulating inflammation. Indeed, changes in miR-223 expression are associated with many infectious diseases (**Figure 2**).

**Table 2:** miR-223 expression in infectious diseases.

Disease	Tissue	Cell type	MiR-223 level	Effect or function	Reference
Chronic hepatitis C (CHC)	Serum	–	Upregulated	Associated with SVR in CHC patients by IFN-based synergy treatment	(91)
CHC	Liver	Hepatocyte	Downregulated	Associated with chronic liver inflammation *via* mediating NF-κB pathway	(92)
Chronic hepatitis B (CHB)	Serum	–	Upregulated	Serve as a biomarker for hepatitis B-induced hepatic damage	(88)
HBV-positive HCC	Serum	HepG2 cell	Downregulated	Viral protein restrains miR-223 expression, thus modulating host immune response	(89)
HCV-positive cirrhosis	Serum	–	Downregulated	Serve as novel non-invasive biomarkers for HCV-associated cirrhosis	(93)
HCV-associated liver fibrosis	Serum	–	Upregulated	Serve as novel non-invasive diagnostic biomarkers for HCV-related liver fibrosis staging	(94)
HBV-associated liver fibrosis	Serum	–	Downregulated	Serve as a non-invasive tool for early diagnosis of HBV-induced liver fibrosis	(95)
HIV-1 infection	–	Resting CD4+T cell	Upregulated	Inhibit viral replication by targeting the 3′ends of HIV-1 messenger RNAs	(100)
HIV-1 infection	–	Macrophage	Upregulated	Promote antiviral activity of IFN-a and IFN-b	(101)
HIV-1 infection	Serum	–	Upregulated	Performed a non-invasive biomarker for early diagnosis early/acute stage of HIV-1 infection	(103)
TB	–	PBMC	Upregulated	Serve as a potential biomarker to discriminate between active TB and latent TB infection	(105)
TB	Blood and lung	Neutrophil	Upregulated	Control the recruitment of neutrophil to the lungs	(73)
TB	–	Macrophage	Upregulated	Inhibit macrophage apoptosis in TB patients	(106)
TB	–	Macrophage	Upregulated	Restrain activation of NF-κB, thus downregulating proinflammatory factors production in macrophages	(108)
TB	–	Macrophage	Upregulated	Inhibit the MMPs production in macrophages under TB infection	(109)
H.pylori-infection	Gastric mucosa	Gastric epithelium cell and neutrophil	Upregulated	MiR-223 is involved in pathogenesis of H.pylori-associated gastritis and may serve as a biomarker for gastritis scores of activity and chronic inflammation	(112)
H.pylori-infection	–	Macrophage	Upregulated	Downregulate pro-inflammatory cytokines, thus inhibiting macrophages activation	(70)
H.pylori-positive gastric cancer	Gastric mucosa	–	Upregulated	Promote cell proliferation and migration, and may take part in course of H.pylori caused chronic inflammation to stomach carcinoma	(113)
Sepsis	Serum	–	Downregulated	Serve as a diagnostic tool for distinguishing between infectious and non-infectious SIRS	(116)
Sepsis	Serum	–	Downregulated	Serve as a novel biomarker for prognosis of sepsis	(117)
Sepsis	Serum	Macrophage	Downregulated at the early stage and up-regulated at the late stage of sepsis	Facilitates IL-4-associated M2-type differentiation of macrophages and decreased clinical scores of sepsis, inhibiting mortality in septic mice.	(66)
Sepsis	Platelet, Plasma and Microparticle	HCAECs	Decreased in Platelet, Elevated in Plasma and Microparticle	Downregulate the expression of ICAM-1 during septic conditions, thus avoiding excessive sepsis-related vascular inflammation	(121)
Sepsis	White blood cell	Lymphocyte	Upregulated	MiR-223 correlated negatively with the percentage of apoptosis in lymphocyte, which may be protective role in sepsis−induced mortality	(122)

### MiR-223 in Viral Hepatitis

Although many viral infections cause hepatitis, including cytomegalovirus, Epstein-Barr virus, influenza, and yellow fever virus, the five hepatitis A–E viruses (HAV–HEV) are the most common causes and are aptly named after the clinical disease. These viruses primarily infect and damage hepatocyte function, and also activate immune cells like macrophages, neutrophils, and T cells, resulting in a cascade of liver inflammation (87). MiR-223 is abundantly expressed in liver and may play a role in viral hepatitis pathology. In 2011, a study revealed that miR-223 levels were higher in serum from hepatitis B patients than healthy people. Interestingly, researchers also found that miR-223 levels were reduced in the liver. Given that miR-223 is highly expressed in hepatic tissue, infection-induced hepatocyte disruption is likely to be the source of miR-223 in circulation and may be used to represent hepatic injury (88). However, another study showed lower miR-223 levels in the serum of HBV-positive HCC patients than healthy controls. HBV-X protein (HBx) was identified as the key regulator of miR-223 and may be responsible for inhibiting miR-223 expression in HepG2 and HepG2.2.15 cells, thus modulating host immune function (89). There is also evidence that HBsAg circulating in HBV carriers is equipped with liver-specific and immune-relevant miRNAs. MiR-223 carried by HBsAg particles appears to aid in intercellular communication, potentially mediating the host immune response. This may explain why a reduction in the level of HBsAg in circulation may indicate that treatment has effectively controlled HBV infection. HBsAg-associated miRNAs may also allow for unintrusive liver-specific miRNA analysis to better understand dynamic changes in miRNA expression in hepatocytes, thus providing a new tool to understand liver physiopathology during viral and non-viral disease (90).

MiR-223 expression is also associated with HCV infection and positive treatment outcomes in humans. Low miR-223 levels were observed in HCV patient serum, while high miR-223 levels were obtained from patients who had reached sustained virologic response (SVR) after treatment. These data indicate that miR-223 levels may show that miRNAs have performed an antiviral function during SVR and/or physiological response to viral clearance. Findings also suggest that different levels of circulating miR-223 correlate with the therapeutic effect and pathological features of liver disease (91). A profiling of miRNA expression in fresh liver biopsies from HCV patients showed that miR-223 levels were lower in chronically infected hepatocytes than normal liver tissue. Notably, low miR-223 levels likely contribute to chronic liver inflammation and subsequent complications by targeting the NF-κB pathway during viral infection (92).

In addition to regulating the early stage of viral infection, miR-223 is also involved in HBV/HCV-related liver fibrosis. Study shows that miR-223 levels are significantly different in serum from HCV-related liver fibrosis patients than healthy people, and may be used as a new non-invasive diagnostic and prognostic marker of HCV-induced liver cirrhosis (93). MiR-223 also contributes to the progression of HCV-related fibrosis. Using the Metavir fibrosis scoring system, higher serum miR-223 levels were associated with significant fibrosis (≥ F2) than no fibrosis or mild fibrosis (F0–F1) (94). A recent study also showed that miR-223 levels differ between stages of liver fibrosis in patients with chronic HBV infection. Using the Scheuer scoring system in HBV-related liver fibrosis, boffins discovered that low levels of miR-223 occurred in the serum as fibrosis progressed from S0–S2 (early fibrosis) to S3–S4 (late fibrosis) (95). These findings indicate that the concentration of miR-223 in circulation is closely associated with virus-associated liver fibrosis staging and may thus serve as a nonintrusive biomarker.

Hepatic fibrosis is a complex and multifactorial pathophysiological progress that is closely linked to chronic inflammatory injury, impairing the balance between production and dissolution of the extracellular matrix (96). It is universally established that immune cells, particularly macrophages, play an essential role in the development or degradation of fibrosis. Macrophages are activated by viruses or other hazardous substances and secrete inflammatory factors and matrix metalloproteinase to accelerate development of HBV/HCV-related liver fibrosis (97). Abnormal miR-223 expression appears to be associated with the pathophysiology of liver cirrhosis given its role in regulating macrophage function.

All of these findings together indicate that miR-223 not only acts as a biomarker but also performs a key role in the pathophysiological progression of viral hepatitis. It is likely to inhibit inflammatory responses caused by hepatitis viruses, supporting the finding that low miR-223 levels are usually associated with chronic inflammatory disorders in the liver. However, the mechanism by which miR-223 controls immune cell activation and regulates hepatitis virus-induced inflammation remains unclear.

### MiR-223 in Human Immunodeficiency Virus (HIV)-1

HIV-1, which primarily infects immune cells, in particular T lymphocytes (CD4+, CD8+), is identified as the causative agent of acquired immunodeficiency syndrome (AIDS), which leads to opportunistic infections and diseases (98, 99). HIV-1 replicates in the cytoplasm with the help of cellular factors. Cells infected with HIV-1 change the expression of multiple cellular miRNAs to promote HIV-1 replication. Given that miRNAs primarily regulate mRNA in the cytoplasm where RNA viruses replicate, it is possible that miRNAs function as antiviral factors by directly targeting and controlling replication of RNA viruses. Indeed, miR-223 levels are closely associated with the susceptibility of immune cells to HIV-1 infection (100). MiR-223 attenuates viral gene expression by directly targeting HIV-1 mRNAs in dormant CD4+ T cells where HIV replication is silenced. Higher levels of miR-223 were observed in resting CD4+T cells than activated CD4+T cells. In addition, reduction of miR-223 alone or in combination with other miRNAs accelerates HIV-1 replication in dormant CD4+ T cells.

Research shows that macrophages stimulated with IFN-α and IFN-β express high levels of miR-223, indicating that this is one of the mechanisms by which macrophages launch an antiviral response (101). A higher level of miR-223 was also observed in monocytes than macrophages, and monocytes are less susceptible to HIV-1 than macrophages, indicating that miR-223 may regulate susceptibility of immune cells (102). A recent study found that miR-223 was expressed differently in individuals with early HIV-1 infection and healthy controls. MiR-223 levels demonstrated 100% sensitivity and specificity (AUC1·00[1·00-1·00]) of the incubation period of HIV-1 and could be used to differentiate between the incubation period of HIV-1 and healthy controls (103). These results suggest that miR-223 may serve as a biomarker for different incubation periods of HIV-1 infection, helping to inform when to begin early antiretroviral therapy and prevent HIV-1 spread. In summary, miR-223 performs a critical role in the progression of AIDS and may be a target for new HIV-1 treatments. Furthermore, miR-223 may serve as a diagnostic tool to aid in determining the incubation period of HIV-1.

### MiR-223 in TB

TB is a chronic disease primarily caused by the mycobacterium TB (MTB) and characterized by long-term fever, dreaminess, expectoration, and emaciation. Bacterial quantity and toxicity, as well as host immune function, play important roles in mediating the occurrence, progression, and prognosis of TB. Dynamic regulation of innate immunity is recognized as a decisive factor in protecting the host from mycobacteria. It is shown that the close cooperation between cytokines and immune cells is essential to anti-TB immunity, of which macrophages, effector CD4^+^T lymphocytes and interferon-γ, are most critical (104). Considering the role miR-223 plays in regulating immune cells, it is possible that miR-223 influences TB pathology.

Recent evidence shows that miR-223 plays an essential part in regulating innate host responses to mycobacterial infection. Earlier studies showed that miR-223 was differentially expressed in the peripheral blood mononuclear cells (PBMCs) of active and latent TB patients, suggesting that miR-223 may regulate TB progression (105). Blood and lung tissue from TB patients were found to contain more miR-223 than healthy controls. Indeed, miR-223 was shown to control migration of polymorphonuclear neutrophils to the lungs by downregulating production of chemokines and proinflammatory cytokines, including CXCL2, CCL3, and IL-6. Although miR-223 KO mice were more susceptible to TB infection and fatal lung inflammation driven by neutrophil recruitment, there were no apparent defects in the T cell response (73). In addition, miR-223 is involved in regulating macrophage apoptosis during TB infection. MiR-223 is abundantly expressed in macrophages from active TB infection patients, preventing expression of forkhead box O3 (FOXO3) and inhibiting macrophage apoptosis (106).

MTB is phagocytosed by and replicates in macrophages following entry into the body. Evidence shows that virulent MTB causes widespread infection by preventing macrophage apoptosis and allowing persistent replication (107). Thus, macrophage apoptosis is one of the mechanisms by which the innate immune system controls TB infection. These observations suggest that miR-223 plays a critical role in mediating the immune response to TB infection by controlling macrophage apoptosis. A higher level of miR-223 was also observed in monocytes and monocyte-derived macrophages from patients with TB than from normal controls. Evidence suggests that a high level of miR-223 may deactivate the NF-κB signaling pathway, impairing expression of proinflammatory cytokines such as IL-1β, IL-6, TNF-α, and IL-12p40, in macrophages (108). A recent study showed that MTB stimulation impacts miR-223, matrix metalloproteinases (MMPs), and brain and muscle ARNT-like1 (BMAL1) levels in macrophages. These cells have high levels of MMPs and miR-223 and a low level of BMAL1 during TB infection. Of note, miR-223 targets the circadian rhythm molecule, BMAL1, controlling MMP expression in macrophages during MTB infection (109). This data further shows how miR-223 mediates expression of circadian clock molecules in macrophages.

In summary, miR-223 is closely associated with the host immune response to MTB. Several studies have defined the regulatory features of miR-223, which appear to interrupt MTB replication and maintain the host’s innate immune. This suggests that miR-223 may be a novel target for MTB treatment. Understanding the mechanism by which miR-223 mediates the immune response to MTB will inform miRNA-based approaches for host-directed therapeutics and vaccines against MTB infections. MiR-22 may also serve as a much-needed biomarker for diagnosing TB and assessing disease progression.

### MiR-223 in *H. pylori* Infection

*H. pylori*, a common bacterium existing in the stomach of half the world’s population, can infect the gastric epithelium and cause chronic gastritis, characterized by high neutrophil and mononuclear cell numbers in the lamina propria (110). Chronic inflammation caused by *H. pylori* is viewed as the most powerful promoter of gastric carcinogenesis and cancer progression (111). Research suggests that miR-223 may play a role in regulating *H. pylori*-related inflammation.

Analysis of the miRNA expression profile in *H. pylori*-infected gastric mucosa using microarray showed that miR-223 was significantly higher in gastric mucosa than healthy mucosa, suggesting that miR-223 is involved in *H. pylori*-associated inflammation (112). Interestingly, miR-223 levels were positively correlated with the density of neutrophil infiltration in the lamina propria, indicating that neutrophils may secrete miR-223. Wang et al. also found that miR-223 was induced by *H. pylori* infection (70), and was negatively correlated with pro-inflammatory cytokines such as IL-6, IL-8, IL-12, and TNF-a, and the activation markers, CD40, CD68, CD80, and CD163, in infected macrophages. High levels of miR-223 in macrophages prevent NF-kB activation by targeting the 3’UTR of IRAK-1 mRNA and reducing gene expression. Thus, miR-223 is a negative regulator of the host immune response to *H. pylori* infection.

A recent investigation found that infection with CagA^+^ *H. pylori* induced miR-223 expression. A higher level of miR-223 was detected in gastric cancer tissues from *H. pylori*-positive patients than *H. pylori*-negative patients (113). NF-κB positively induces miR-223 by binding to its promoter region, which may explain why CagA^+^ *H. pylori* induces miR-223 expression. MiR-223 targets ARID1A, promoting cell proliferation and migration, suggesting that the NF-κB/miR-223-3p/ARID1A axis may promote *H. pylori*-induced chronic inflammation associated with gastric carcinoma.

Taken together, these results indicate that miR-223 plays a critical role in the host immune response to *H. pylori*, bridging *H. pylori*, chronic inflammation, and the development of precancerous lesions. These findings indicate that miR-223 may serve as a novel tool for the diagnosis and treatment of *H. pylori*-induced chronic inflammation during gastric carcinoma.

### MiR-223 in Sepsis

Sepsis is primarily caused by pathogenic microorganisms and is characterized by an excessive systemic inflammatory response (114). By impairing heart, liver, lung, and kidney tissue, sepsis is regarded as the biggest cause of mortality in intensive care units (115). An excessive systemic inflammatory response and multiple inflammatory cytokines cause the organ dysfunction observed during sepsis.

Emerging evidence suggests that miR-223 may be a new biomarker to aid in the diagnosis of sepsis (116). Serum collected from 50 patients with sepsis, 30 patients with systemic inflammatory response (SIRS), and 20 healthy controls, was assessed using quantitative PCR to examine seven miRNAs associated with inflammation and infection. MiR-223 levels were significantly lower in serum from sepsis patients than from both SIRS patients and healthy people. Interestingly, miR-223 levels were similar between non-infectious SIRS patients and healthy people, suggesting that miR-223 may serve as a diagnostic biomarker used to distinguish between infectious and non-infectious SIRS. Another study collected serum from 214 sepsis patients(117 survivors and 97 non-survivors) and measured miR-223 levels. Results showed that a lower level of miR-223 was present in the serum of non-surviving than surviving sepsis patients (117). These findings indicate that miR-223 not only serves as a diagnostic marker of sepsis but may also affect its pathophysiological processes, especially regulation of organ dysfunction caused by excessive systemic inflammation. A subsequent study indicated that miR-223^-/-^ mice had more deteriorative sepsis-associated heart dysfunction, stronger inflammatory responses, and higher mortality than wild-type mice (118). Cardiomyocytes from the hearts of miR-223^-/-^ mice showed higher levels of TNF-α and IL-1β, indicating that miR-223 deficiency exacerbates the cardiomyocyte-specific immune response during sepsis.

Another study reported that miR-223 played a protective role in sepsis-induced myocardial dysfunction (119). In this investigation, cecal ligation and puncture (CLP) was used to establish a sepsis model in mice. MiR-223-null mesenchymal stem cells (MSCs) and WT-MSCs were injected into specific mice to assess whether CLP-triggered harm could be alleviated. As expected, mice receiving miR-223-null-MSCs suffered more severe cardiac dysfunction, increased apoptosis, and inflammatory responses, suggesting that miR-223 plays an important role in modulating MSC-induced cardio-protection during sepsis. At the molecular level, MSC-derived exosomal miR-223 significantly suppressed the activation of macrophages in the septic model. Exosomal miR-223 was negatively correlated with production of pro-inflammatory cytokines like TNF-α, IL-1β, and IL-6, by suppressing Sema3A and STAT3 protein expression in macrophages. Two additional studies identified a critical function for macrophages during sepsis. High miR-223 levels were shown to facilitate M2 macrophage-polarization during LPS stimulation, which is likely associated with interference of glycolysis by reducing HIF-1α. Of note, injection of miR-223-overexpressed macrophages that were pretreated with IL-4, alleviated sepsis symptoms in the LPS model (120). In another study, researchers found that miR-223 was expressed at high levels during the early stage and low levels during the late stage of sepsis in macrophages, and miR-223 deficiency worsened disease and contributed to high mortality.

Interestingly, miR-223 levels were positively correlated with M2 macrophage numbers observed during sepsis. Indeed, miR-223 was a critical factor in regulating IL-4-induced M2 differentiation of macrophages by controlling Nfat5 and Rasa1 protein production, thus modulating the pathophysiology of sepsis (66). In addition, miR-223 is involved in human coronary artery endothelial cell (HCAECs) activation during septic conditions. Platelet-derived microparticles (PMPs) carry miR-223 to endothelial cells and maintain intercellular adhesion molecule-1 (ICAM-1) at low levels under septic conditions, thus avoiding excessive sepsis-associated vascular inflammation (121). An additional study showed that a higher level of miR-223 existed in septic patients than healthy controls. More miR-223 was found in the survivor group than the non-surviving group, associating miR-223 levels with the risk of death during septic conditions.

Studies indicate that the level of miR-223 is negatively associated with lymphocyte apoptosis by targeting FOXO1 during sepsis (122). Thus, miR-223 appears to be a prognostic biomarker that could be used to formulate a therapeutic plan that is based on the severity of sepsis. Patients with a low level of miR-223 appear to have a higher risk of mortality. In addition, miR-223 is involved in the pathophysiological processes of sepsis, regulating organ dysfunction caused by excessive systemic inflammatory response. Therefore, miR-223 may serve as a possible target for effective therapies during sepsis. However, due to the small sample size of these studies, the reliability and accuracy of miR-223 as a diagnostic tool for sepsis severity requires further study. In addition, the mechanism and effectiveness of miR-223 as a treatment for sepsis needs further exploration.

## Conclusions and Perspectives

In summary, miR-223 plays an important role in the immune response, regulating multiple processes from myeloid differentiation to neutrophil, macrophage, and DC function. Many studies have shown that miR-223 is dysregulated during inflammatory diseases, and can target several immune-related genes to mediate inflammation. During many infectious diseases, including viral hepatitis, HIV, TB, and sepsis, miR-223 expression was altered (down or upregulated) and found to play an essential role in maintaining the balance of innate immunity to avoid excessive inflammation and tissue injury. In addition, miR-223 is involved in inhibiting virus replication and regulating inflammation-induced carcinogenesis.

Considering its important role in mediating immune cell function and infectious pathophysiology, miR-223 is identified as a promising biomarker and therapeutic tool for the diagnosis and therapy of infectious diseases. However, further investigation is needed to verify the accuracy, reliability, and prognostic and therapeutic potential of miR-223. First, circulating miR-223 has distinct expression profiles during different infectious diseases, indicating that large-sample clinical studies are required to further determine its ability to accurately diagnose infection. In addition, miR-223 not only regulates gene expression in an intracellular manner to mediate myeloid cell responses to infection but can also be processed into exosomes and used as an intercellular communication tool. Thus, miR-223-based therapy using miR-223 analogues or cells expressing high levels of miR-223 may be an exciting option for infectious disease treatment. Future studies should assess how to selectively and accurately target miR-223-derived drugs to infection sites. In addition, the safety and efficacy of miR-223-based treatment need to be further evaluated in larger animals and clinical trials. Finally, studies must further elucidate potential miR-223 targets and determine how it correlates with irritation and most cancers, improving the current understanding of miR-223 biology and contributing to safer and more precise treatment for infectious diseases.

In summary, while already used as a biomarker in numerous inflammatory disorders, the precise mechanism and therapeutic capacity of miR-223 should be explored more comprehensively to assess how it may be used to improve treatments for a wide range of inflammatory disorders.

## Author Contributions

Designed and wrote the manuscript: SY and QW. Revised the content: YYC and SZ. Modified the language: XZ and FS. Draw the images: YJC. All authors contributed to the article and approved the submitted version.

## Funding

This work was supported by the National Natural Science Foundation of China (Grant No.81570428), Key Support Project of Health Commission of Hubei Province (Grant No. WJ2019Z012), and Guiding Fund of Renmin Hospital of Wuhan University (Grant No. RMYD2018Z07).

## Conflict of Interest

The authors declare that the research was conducted in the absence of any commercial or financial relationships that could be construed as a potential conflict of interest.

## Publisher’s Note

All claims expressed in this article are solely those of the authors and do not necessarily represent those of their affiliated organizations, or those of the publisher, the editors and the reviewers. Any product that may be evaluated in this article, or claim that may be made by its manufacturer, is not guaranteed or endorsed by the publisher.
